# Impacts of Land Use and Water Quality on Macroinvertebrate Diversity Under Human Disturbance in the Lake Chaohu Basin, China

**DOI:** 10.1002/ece3.72415

**Published:** 2025-10-30

**Authors:** Bingling Chen, Qing Ji, Youru Yao, Zhiming Zhang, Yuesheng Lin

**Affiliations:** ^1^ Key Laboratory of Earth Surface Processes and Regional Response in the Yangtze‐Huaihe River Basin, School of Geography and Tourism Anhui Normal University Wuhu China

**Keywords:** Lake Chaohu Basin, land use types, macroinvertebrates, Partial Least Squares Path Modeling, water quality

## Abstract

Freshwater ecosystems play a critical role in sustaining biodiversity, but with increasing anthropogenic disturbances in recent years, issues such as water degradation and biotic community decline have become increasingly severe. Although existing research has explored the impact of water quality factors and land use on macroinvertebrate communities, the specific mechanisms by which human activities indirectly influence macroinvertebrate diversity by altering the aquatic environment are still underexplored. Therefore, this study, on the basis of land use, aquatic environmental, and macroinvertebrate survey data, employed a Partial Least Squares Path Model (PLS‐SEM) to elucidate the mechanisms by which land use and water quality factors jointly drive changes in macroinvertebrate communities. Our results demonstrate that macroinvertebrate community structure varied significantly among disturbance levels, with biodiversity indices increasing progressively as disturbance intensity lessened. Specifically, pollution‐tolerant taxa dominated in high disturbance areas, *Bellamya* and *Alocinma* were predominant in moderate disturbance areas, whereas sensitive species were dominant in low disturbance areas. Land use explained 11.1% of the variation in diversity, about 2.6 times that of water quality factors (4.3%), indicating clear differences in their driving effects on macroinvertebrate diversity, as shown by variance partitioning analysis (VPA). According to the PLS‐SEM results, built‐up land, as the main negative factor, exerted combined effects on diversity both directly and indirectly by causing water quality deterioration. Water quality factors exhibited spatially differentiated effects: ammonia nitrogen and total nitrogen strongly inhibited diversity in high and moderate disturbance areas, whereas in low disturbance areas, total hardness and water temperature emerged as the primary positive drivers. This study elucidates the dynamic response mechanisms of macroinvertebrate communities to changes in land use and water quality, offering new perspectives for freshwater ecosystem health assessments and biodiversity conservation.

## Graphical abstract

Our study revealed that human activities drive changes in macroinvertebrate diversity through the combined effects of land use and water quality. The results showed that sensitive taxa (*Semisulcospira cancelata*) dominated in low disturbance areas, *Bellamya* (*Bellamya aeruginosa*) and *Alocinma* (*Alocinma longicornis*) were predominant in moderate disturbance areas, whereas tolerant taxa ( *Physa acuta* ) dominated in high disturbance areas. Our study reveals the mechanism by which human disturbances influence community diversity through the combined effects of land use and water quality, providing a reference for freshwater ecosystem health assessment and biodiversity conservation.

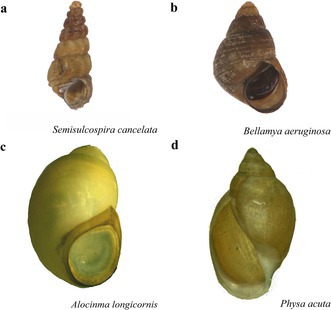

## 1 Introduction

Freshwater ecosystems, under the combined pressures of global climate change and human activities, are experiencing ongoing water quality deterioration and significant biodiversity loss (Albert et al. 2021; Piggott et al. 2015; Reid et al. 2019). In response to this crisis, the past few decades have seen the gradual development of ecosystem assessment frameworks focusing on aquatic organisms—including fish, large aquatic plants, diatoms, and macroinvertebrates—which have become a primary focus in both scientific research and management practices (Birk et al. 2012; Wu et al. 2018). Among them, macroinvertebrates play a crucial role in maintaining biodiversity and ecosystem stability in aquatic environments (Tampo et al. 2021; Wang, Wang, Xia, et al. 2021; Wang, Wang, Lin, et al. 2021). Therefore, studying the characteristics of freshwater macroinvertebrate communities and their responses to environmental changes has become a crucial goal for restoring and maintaining the health of freshwater ecosystems (Ciucure et al. 2023).

Benthic macroinvertebrates constitute a vital part of freshwater ecosystems and are broadly distributed across rivers, lakes, and wetlands. They play a crucial role in material cycling and energy flow through processes such as organic matter decomposition, sediment disturbance, and energy transfer (Devine and Vanni 2002). Compared with other aquatic taxa, macroinvertebrates are characterized by high species diversity, limited dispersal ability, and long life cycles (Hajializadeh et al. 2020; Liu et al. 2023). Because of their sensitivity to environmental changes, habitat at the water bottom, limited activity range, and ease of sampling, macroinvertebrate community structure changes can effectively indicate water pollution levels, thus serving as key biological indicators for global freshwater assessments (Moi et al. 2022; Pillay 2022).

Multiple factors influence the structure of benthic macroinvertebrate communities, with the combined effects of land use and water quality recognized as primary drivers (Fergus et al. 2023; Lu et al. 2022; Munyai et al. 2024). Studies have shown that changes in land use can significantly alter aquatic environmental characteristics, thereby affecting benthic macroinvertebrate communities. For example, agricultural practices contribute to elevated inputs of nitrogen and phosphorus, resulting in the eutrophication of aquatic systems (Bohn et al. 2024). Conversely, urban land use is strongly linked to increases in biochemical oxygen demand (Lee et al. 2020). These changes can further affect key water quality parameters such as temperature, dissolved oxygen, and conductivity, thereby filtering benthic macroinvertebrate species adapted to different environmental conditions, ultimately influencing community composition and functional diversity (Feng et al. 2024; Munyai et al. 2025; Ni et al. 2024). Although many studies have examined the influence of land use and water environmental factors on macroinvertebrate community structure, the distinct pathways through which these factors operate across different disturbance gradients remain to be explored in greater depth (Fierro et al. 2017; Ma et al. 2023; Park et al. 2025; Quinn et al. 1997; Whiles et al. 2000).

On the basis of the above‐identified gaps in current research, this study aims to systematically assess the combined effects of land use and water quality on macroinvertebrate diversity under human disturbance, and to explore the pathways through which these effects occur, thereby providing a scientific basis for the precise protection and management of watershed ecosystems. Accordingly, the specific aims of this research are as follows: (1) exploring macroinvertebrate community structure and its associations with water quality under anthropogenic disturbance; (2) investigating the influence of land use and water quality on macroinvertebrate diversity across varying levels of disturbance, and elucidating the underlying mechanisms through path analysis. The findings provide a theoretical foundation for the conservation and sustainable management of aquatic ecosystems.

## 2 Materials and Methods

### 2.1 Study Area and Sampling Site Design

**FIGURE 1:**
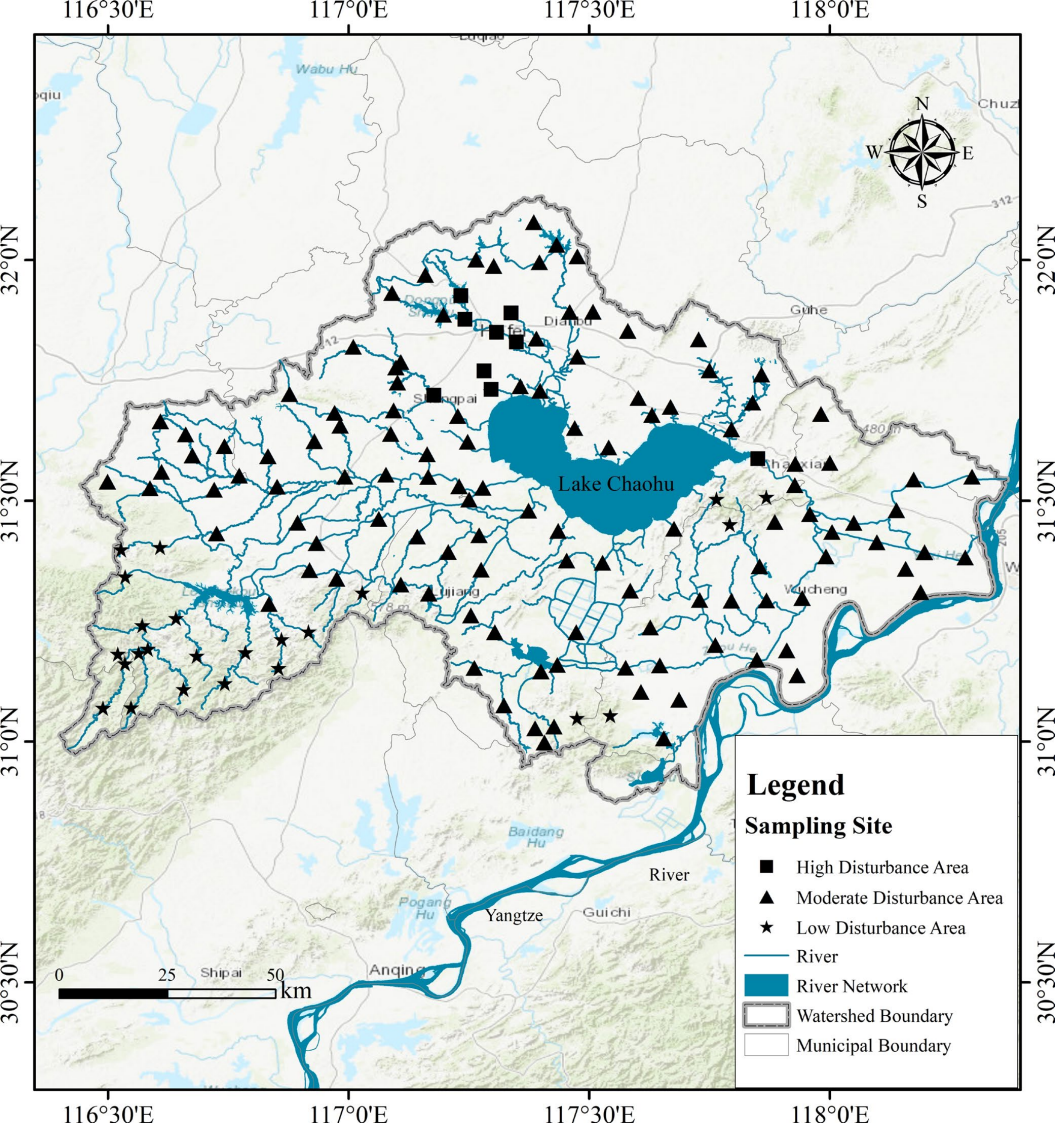
Distribution of sampling sites in the Lake Chaohu Basin.

The Lake Chaohu Basin (30°52′~32°07′ N, 116°23′~118°22′ E) is located in central Anhui Province; it belongs to the middle‐lower reaches of the Yangtze River system (Figure 1). As one of China's five largest freshwater lakes, it encompasses a watershed area of approximately 13,400 km^2^, with a length of 54.5 km from east to west, a width of 21.0 km from north to south, and an average water depth of 2.69 m (Gao et al. 2016). The basin is characterized by a subtropical humid monsoon climate, with a mean annual temperature of 16°C and an average annual precipitation of 1100 mm (Huang et al. 2013). The topography of the Lake Chaohu Basin slopes from west to east, and from north to south, with elevated hilly terrain in the southwest and low‐lying plains in the southeast. Because of intensified human activities, land use patterns in the Lake Chaohu Basin have changed, with cropland and wetlands gradually being converted to built‐up land, especially because of the expansion of Hefei City and frequent industrial and agricultural activities.

### 2.2 Macroinvertebrate Sampling Processing

In the Lake Chaohu Basin, this study used DEM data with a spatial resolution of 30 m (https://www.resdc.cn/Default.aspx) and delineated catchment boundaries through ArcGIS hydrological analysis. Using a grid‐based point allocation method, 150 sampling sites were established near the outlets of the catchments to conduct surveys on land use types, river water quality, and the species composition and abundance of macroinvertebrates. In April 2013, a total of 150 macroinvertebrate samples were collected from these sampling sites. For benthic macroinvertebrates, a D‐frame net was used to sample ten 30 cm × 50 cm quadrats per site on the basis of habitat type proportions, and the samples were combined into one composite sample. Macroinvertebrates in the river channel were sampled using a 1/16 m^2^ Peterson grab with six replicates per site. The samples were combined and filtered in the field with a 60 mm nylon sieve, and the residues were placed in plastic jars with 7% formalin for laboratory analysis. Macroinvertebrates were identified and counted under microscopes and dissecting scopes, with taxa identified to the lowest possible taxonomic level, down to species in this study (Tang 2006; Wang 2002). Excess moisture on the surface of macroinvertebrates was removed using filter paper, after which the specimens were weighed with an electronic balance. On the basis of the sampling area of each site, the final results were standardized and expressed as density (ind./m^2^) and biomass (g/m^2^).

### 2.3 Water Environment Factor Analysis

Water samples were collected at 0.5 m below the surface using a 2.5 L plexiglass water sampler and preserved at below 4°C for laboratory analysis (Hou et al. 2018; Wu et al. 2013). In situ measurements of pH, electrical conductivity (EC), water temperature (WT), and turbidity (Turb) were performed using a multiparameter water quality probe (YSI 6600V2). Ammonia nitrogen (NH_3_‐N), nitrate nitrogen (NO_3_‐N), and phosphate (PO_4_‐P) concentrations were determined using a microflow injection analyzer. Water samples were collected, frozen, and transported back to the laboratory, where total nitrogen (TN, mg/L), dissolved organic carbon (DOC, mg/L), permanganate index (COD_Mn_, mg/L), total hardness (TH, mg/L), and alkalinity (mg/L) were measured following the *Standard Methods for the Examination of Water and Wastewater* (The State Environmental Protection Administration 2002). To ensure the consistency between biological and environmental data, water samples were collected concurrently with macroinvertebrate sampling at the same locations and time during the 2013 field survey.

### 2.4 Land Use Data

The land use information within the catchments of sampling sites was extracted from the 2013 national land use satellite remote sensing data with a spatial resolution of 30 m (http://doi.org/10.5281/zenodo.4417809). Cropland, forestland, and built‐up land were identified as the main land use categories in the sampling areas. Using ArcGIS 10.8, we first calculated the area of different land use types within each watershed and computed their relative proportions (see Table S1). Using the proportions of cropland, forest, and built‐up land as indicators of human disturbance, and following previous studies (Li, Zhang, Altermatt, et al. 2023; Li, Zhang, Zhang, et al. 2023), we applied the *K*‐means clustering method to classify the sampling sites. The optimal number of clusters was determined to be *k* = 3 on the basis of the silhouette coefficient, resulting in three groups (see Table S2). According to the land use characteristics of each group, we labeled them as low disturbance areas (LDA, with forest cover typically exceeding 70%), moderate disturbance areas (MDA, dominated by cropland with cropland proportion typically above 80%), and high disturbance areas (HDA, with built‐up land proportion typically exceeding 65%). These groupings were then used as the basis for subsequent analyses.

### 2.5 Data Analysis

#### 2.5.1 Biodiversity Indices Calculation

The biotic index included the Shannon–Wiener diversity index (*H*′) (Shannon 1948), the Simpson's dominance index (*D*) (Simpson 1949), the Margalef richness index (*d*) (Margalef 1958), and the Pielou evenness index (*J*) (Pielou 1966). These indices collectively reflect changes in species richness and the evenness of relative abundance under environmental disturbance.

#### 2.5.2 Statistical Analysis

To ensure the suitability of the statistical methods, we first conducted a Kolmogorov–Smirnov test for normality on the water quality. The results showed that the data were normally distributed but exhibited heterogeneity of variance; therefore, a Kruskal–Wallis non‐parametric test was applied to analyze the differences in 12 environmental factors and to compare macroinvertebrate diversity across different disturbance zones. SIMPER (Similarity Percentage analysis) was used to assess the average contribution of dominant species to within‐group similarity in each region (Clarke and Gorley 2006; Clarke 1993). The analysis was based on the Bray–Curtis similarity index and performed using species density data. The species with the highest cumulative contribution in each region were considered key characteristic taxa and applied in the following canonical correspondence analysis (CCA). We employed CCA to explore the relationship between macroinvertebrate diversity and water environmental variables. Environmental variables were selected using a forward selection procedure (Monte Carlo permutation test, *p* < 0.05). Species data were square‐root‐transformed, and environmental variables (except pH) were log‐transformed.

In this study, VPA was used to quantify the contributions of land use and water quality factors to macroinvertebrate diversity, and a PLS‐SEM was developed to explore how land use and water quality factors affect macroinvertebrate diversity (Chang et al. 2025; Zhao et al. 2024). VPA provides a direct measure of variance attribution, whereas PLS‐SEM captures the hierarchical and mediating effects among variables, making the two approaches complementary in addressing the research objectives. Moreover, PLS‐SEM breaks through various constraints of traditional Structural Equation Modeling (SEM), especially its reliance on multivariate normality assumptions, and demonstrates greater flexibility in handling small sample sizes (Hair et al. 2021; Kock 2016). PLS‐SEM consists of two sub‐models: the measurement model and the structural model. The measurement model captures the associations between latent constructs and their observed variables, which are expressed through weights. The structural model illustrates the interrelationships among latent variables, indicated by path coefficients. The model's overall explanatory and predictive performance was evaluated using the Goodness‐of‐Fit (GOF) index, which can be interpreted as follows: GOF < 0.25 indicates a weak model fit; 0.25 < GOF < 0.36 suggests a moderate fit; and GOF > 0.36 indicates a strong model fit (Wang, Liu, Wang, et al. 2021).

The Kolmogorov–Smirnov and Kruskal–Wallis non‐parametric tests, SIMPER, DCA, VPA, and the PLS‐SEM were all conducted on the R platform. CCA was conducted with the CANOCO 5.0 software package. Macroinvertebrate diversity indices were calculated using PAST version 3.0.

## 3 Results

### 3.1 River Water Quality Characteristics in the Lake Chaohu Basin

The Kruskal–Wallis test revealed that, except for pH, water environmental factors in the Lake Chaohu Basin exhibited significant differences across the different disturbance areas (Table 1). In the HDA, concentrations of NH_3_‐N, TN, PO_4_‐P, and DOC were significantly higher than those in the LDA and MDA (*p* < 0.05), whereas Turb and Alkalinity were elevated in both MDA and HDA. The MDA showed notably higher concentrations of COD_Mn_ and TH. NO_3_‐N levels were significantly higher in the LDA compared to the MDA and HDA. The impact of disturbance areas on WT was limited, with only minor differences observed among the three areas.

**TABLE 1:** Comparison of river water quality characteristics across disturbance areas in the Lake Chaohu Basin (mean ± standard deviation; COD_Mn_: permanganate index; DOC: dissolved organic carbon; EC: electrical conductivity; HDA: high disturbance areas; LDA: low disturbance areas; MDA: moderate disturbance areas; NH_3_‐N: ammonia nitrogen; NO_3_‐N: nitrate; PO_4_‐P: phosphate; TH: total hardness; TN: total nitrogen; Turb: turbidity; WT: water temperature).

Variable	HDA	MDA	LDA	*p*
pH	8.32 ± 1.26	7.85 ± 0.44	8.03 ± 0.56	> 0.05
EC (μs/cm)	292.222 ± 133.576	251.282 ± 306.550	107.250 ± 90.267	< 0.05
NH_3_‐N (mg/L)	12.220 ± 9.494	1.717 ± 4.265	0.295 ± 0.151	< 0.05
NO_3_‐N (mg/L)	0.170 ± 0.283	0.196 ± 0.298	0.716 ± 0.692	< 0.05
TN (mg/L)	14.187 ± 9.519	3.101 ± 7.211	2.052 ± 0.900	< 0.05
PO_4_‐P (mg/L)	1.409 ± 1.864	0.174 ± 0.623	0.023 ± 0.012	< 0.05
DOC (mg/L)	10.163 ± 3.894	6.141 ± 2.772	3.132 ± 1.110	< 0.05
COD_Mn_ (mg/L)	5.724 ± 3.002	6.060 ± 6.276	3.992 ± 2.737	< 0.05
Turb (mg/L)	25.856 ± 19.257	19.023 ± 25.009	5.929 ± 9.537	< 0.05
WT (°C)	20.283 ± 4.136	18.673 ± 3.236	16.262 ± 2.711	< 0.05
TH (mg/L)	105.781 ± 18.117	109.804 ± 53.854	63.946 ± 72.862	< 0.05
Alkalinity (mg/L)	92.964 ± 49.897	51.845 ± 29.679	29.797 ± 21.267	< 0.05

### 3.2 Characteristics of Macroinvertebrate Communities

#### 3.2.1 Density and Biomass

A total of 154 species of macroinvertebrates were collected in the Lake Chaohu Basin, representing 3 phyla, 6 classes, 21 orders, 69 families, 110 genera. Among them, *Arthropoda* were the highest species richness group, comprising 112 species (72.7% of the total), classified into 2 classes, 12 orders, 52 families, and 79 genera. *Mollusca* were the second most abundant group, with 30 species (19.5%), belonging to 2 classes, 5 orders, 11 families, and 21 genera. *Annelida* were the least represented, with 12 species (1.3%), distributed across 2 classes, 6 orders, 7 families, and 10 genera. The most frequently encountered species were *Bellamya aeruginosa* (occurrence rate: 72.67%) and *Radix swinhoei* (occurrence rate: 54%).

Significant variations in species density and biomass were observed across the three disturbance areas (Table 2). *Oligochaeta* contributed the most to the overall density (94.8%), whereas *Gastropoda* accounted for the highest proportion of total biomass (66.5%). The highest density of *Oligochaeta* was recorded in the LDA (1696.65 ± 1233.99 ind./m^2^), whereas the lowest was found in the HDA (0.87 ± 0.31 ind./m^2^). Biomass of *Gastropoda* (26.05 ± 4.56 g/m^2^) and *Bivalvia* (8.89 ± 2.17 g/m^2^) was significantly higher in the MDA than in the other areas, mainly attributed to large snails such as *Bellamya aeruginosa*. The density and biomass of *Crustacea* did not differ significantly among the three areas (*p* < 0.05).

**TABLE 2:** Density and biomass of macroinvertebrates across disturbance areas in the Lake Chaohu Basin (mean ± standard deviation).

Index	Taxonomic group	HDA	MDA	LDA	*H*	*p*
Density (individuals/m^2^)	Bivalvia	2.58 ± 1.33	3.25 ± 0.78	0.12 ± 0.12	8.26	0.016
	Oligochaeta	0.87 ± 0.31	1031.47 ± 881.04	1696.65 ± 1233.99	10.19	0.006
	Insect	7.80 ± 1.47	30.58 ± 20.95	9.44 ± 4.39	6.02	0.049
	Gastropoda	13.58 ± 3.91	34.18 ± 10.69	36.72 ± 32.13	8.07	0.018
	Hirudinea	0.16 ± 0.07	2.10 ± 0.93	0.37 ± 0.37	8.47	0.014
	Crustacea	2.74 ± 1.05	5.77 ± 1.41	0.45 ± 0.34	2.13	0.345
	Total density	27.73 ± 4.14	1107.38 ± 910.94	1743.75 ± 1226.54	/	/
Biomass (g/m^2^)	Bivalvia	0.99 ± 0.63	8.89 ± 2.17	0.02 ± 0.02	13.64	0.001
	Oligochaeta	0.02 ± 0.01	2.94 ± 2.09	3.70 ± 2.52	7.25	0.027
	Insect	0.14 ± 0.03	0.13 ± 0.05	0.14 ± 0.06	9.29	0.010
	Gastropoda	5.18 ± 2.08	26.05 ± 4.56	4.79 ± 2.42	20.81	*p* < 0.001
	Hirudinea	0.01 ± 0.01	0.03 ± 0.01	0.02 ± 0.02	7.64	0.022
	Crustacea	0.41 ± 0.18	0.67 ± 0.17	0.05 ± 0.05	2.27	0.322
	Total biomass	6.73 ± 2.20	38.72 ± 5.59	8.72 ± 2.84	/	/

Overall, macroinvertebrate density and biomass varied markedly among disturbance levels: density followed the pattern HDA (27.73 ind./m^2^) < MDA (1107.38 ind./m^2^) < LDA (1743.75 ind./m^2^), whereas biomass followed the pattern HDA (6.73 g/m^2^) < LDA (8.72 g/m^2^) < MDA (38.72 g/m^2^).

#### 3.2.2 Dominant Species

**FIGURE 2:**
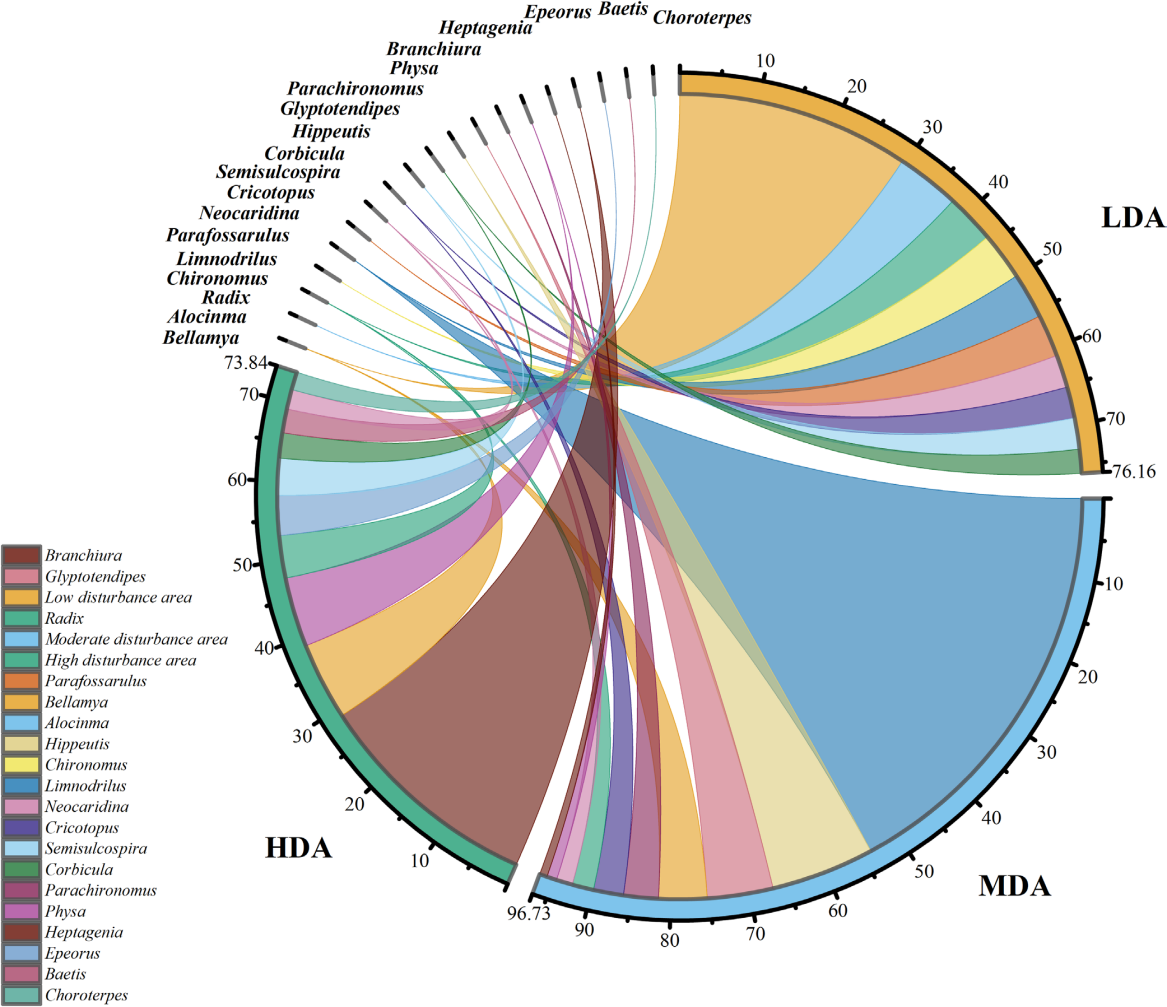
Dominant macroinvertebrate taxa and their contribution rates (%) to community composition in different disturbance areas on the basis of SIMPER. Colors represent taxa as follows: *Alocinma* (sky blue), *Baetis* (rose red), *Bellamya* (goldenrod), *Branchiura* (dark brown), *Chironomus* (lemon yellow), *Choroterpes* (teal green), *Corbicula* (forest green), *Cricotopus* (indigo), *Epeorus* (light steel blue), *Glyptotendipes* (dusty rose), *Heptagenia* (dark brown), *Hippeutis* (pale pink), *Limnodrilus* (medium blue), *Neocaridina* (orchid), *Parachironomus* (maroon), *Parafossarulus* (rust orange), *Physa* (plum), *Radix* (jade green), and *Semisulcospira* (light sky blue).

Our results indicated that the dominant taxa contributing to the differences among disturbance areas differed markedly (Figure 2). In the HDA, *Limnodrilus*, *Hippeutis*, and *Glyptotendipes* showed relatively high contributions. In the MDA, *Mollusca*, such as *Bellamya* and *Alocinma* were the main contributing taxa. In the LDA, *Heptagenia* and *Bellamya* were the dominant contributors.

Overall, *Bellamya, Radix, and Neocaridina* were dominant taxa across all three disturbance areas. *Limnodrilus and Cricotopus* were mainly present in the MDA and HDA, whereas *Semisulcospira* and *Corbicula* exhibited higher abundance in the LDA and MDA. Other species were dominant only in a single disturbance category.

#### 3.2.3 Biodiversity Indices

**FIGURE 3:**
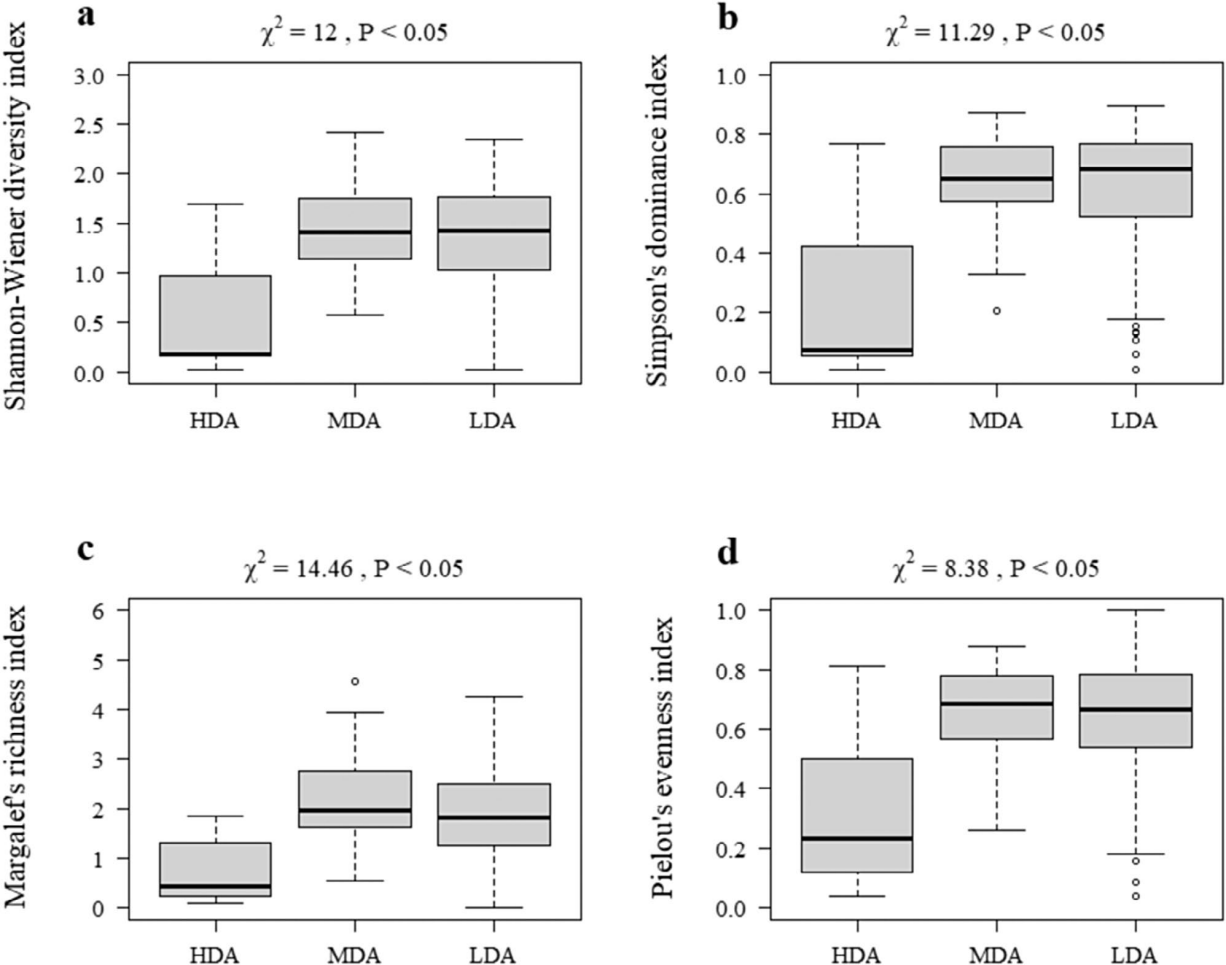
Boxplots of the Shannon–Wiener Diversity Index (a), Simpson's Dominance Index (b), Margalef's Richness Index (c), and Pielou's Evenness Index (d) across disturbance areas in the Lake Chaohu Basin. HDA, high disturbance areas; LDA, low disturbance areas; MDA, moderate disturbance areas.

Different macroinvertebrate diversity indices showed significant responses to varying disturbance areas (Figure 3, *p* < 0.05). The Shannon–Wiener diversity index, Pielou's evenness index, Margalef's richness index, and Simpson's dominance index ranged from 0.03 to 2.42 (1.34 ± 0.57), 0.04 to 1.00 (0.63 ± 0.19), 0.10 to 4.55 (1.88 ± 0.75), and 0.01 to 0.90 (0.60 ± 0.21), respectively. All four indices were lowest in the HDA, with values of 0.54 ± 0.21, 0.33 ± 0.10, 0.73 ± 0.23, and 0.25 ± 0.10, respectively. In contrast, the values of all four indices were relatively higher in the LDA and MDA, with the LDA showing values of 2.24 ± 0.19, 0.65 ± 0.03, 1.48 ± 0.10, and 0.63 ± 0.04, whereas the MDA showed values of 1.89 ± 0.09, 0.64 ± 0.02, 1.37 ± 0.05, and 0.61 ± 0.02.

### 3.3 Relationships Between Macroinvertebrate Communities and Water Quality Variables

**FIGURE 4:**
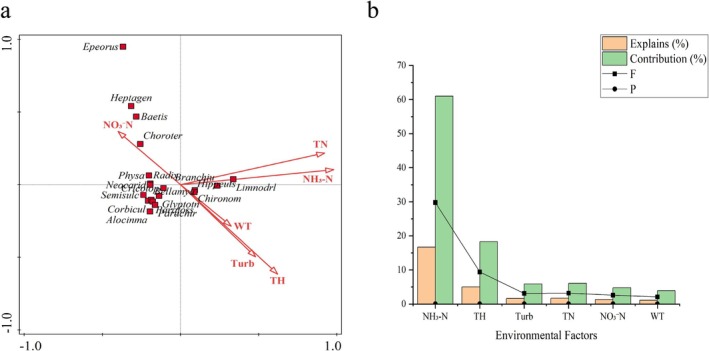
CCA analysis of macroinvertebrate communities and environmental variable importance in the Lake Chaohu Basin. (a) The CCA ordination plot illustrates the direction and strength of major environmental factors influencing the distribution of macroinvertebrate communities. (b) The explanatory power (Explains, %) and contribution (Contribution, %) of each environmental variable to community variation, accompanied by *F*‐values and *p*‐values from permutation tests. Arrows represent environmental factors in the plot, and their quadrant placement indicates the positive or negative correlation with the ordination axes. The direction and length of the arrows reflect the direction and strength of the relationship between environmental factors and community distribution.

According to the CCA results (Table 3), the eigenvalues of the first two ordination axes were 0.67 and 0.28, accounting for 17.35% and 7.31% of the total variance in the macroinvertebrate community, respectively. Given the large number of water environmental variables, forward selection was used to identify key environmental factors, and their significance was tested using Monte Carlo permutation tests. The forward selection results indicated that 11 water environmental factors significantly affected the structure of the macroinvertebrate community. After removing six non‐significant variables, the cumulative explanatory power of the species–environment data increased from 92.69% to 98.03%. According to the results of the Monte Carlo permutation test (Figure 4b), the dominant environmental factors influencing the macroinvertebrate community structure in the Lake Chaohu Basin were NH_3_‐N, TH, Turb, TN, NO_3_‐N, and WT (*p* < 0.05). Among these, NH_3_‐N and TH exhibited relatively high explanatory power, indicating that they are key drivers shaping macroinvertebrate community characteristics, whereas Turb, TN, NO_3_‐N, and WT showed comparatively lower explanatory power.

**TABLE 3:** CCA analysis of macroinvertebrate communities and environmental variables.

Item	Axis1	Axis2	Axis3	Axis4
Eigenvalue	0.67	0.28	0.06	0.03
Species‐environment correlation	17.35	24.66	26.11	26.88
Cumulative percentage of species data variance	0.92	0.65	0.44	0.38
Cumulative percentage of species‐environment variance	63.26	89.92	95.20	98.03

CCA illustrated how macroinvertebrate community structure responds to variations in water quality factors (Figure 4a). Specifically, the distribution of *Hippeutis*, *Branchiura*, and *Limnodrilus* was positively correlated with TN and NH_3_‐N, indicating their high pollution tolerance and preference for nutrient‐rich waters, whereas 12 aquatic insect taxa including *Chironomus*, showed the opposite trend. Taxa such as *Epeorus, Baetis, Rhithrogena*, and *Heptagenia* preferred environments characterized by low nutrients, low Turb, and low WT, and showed a positive correlation with NO_3_‐N.

### 3.4 Path Analysis of the Effects of Environmental Factors on Macroinvertebrate Diversity Across Disturbance Areas

**FIGURE 5:**
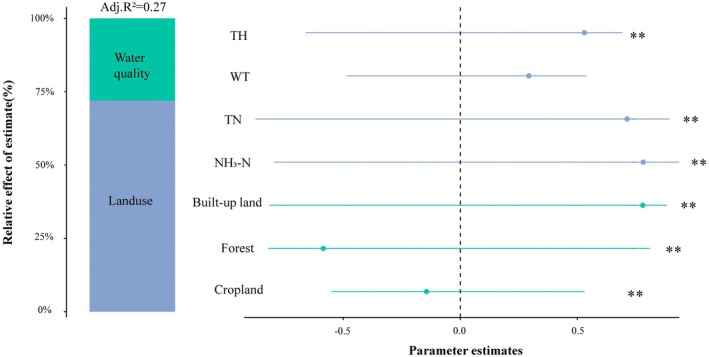
VPA of land use and water quality factors on macroinvertebrate diversity. The fractions represent the proportion of variance in macroinvertebrate diversity explained by the predictors: Green indicates the variance explained uniquely by land use, blue indicates the variance explained uniquely by water quality, and the overlap indicates the shared contribution of both. Residuals represent the unexplained variance not accounted for by the selected variables. *p < 0.01.

**FIGURE 6:**
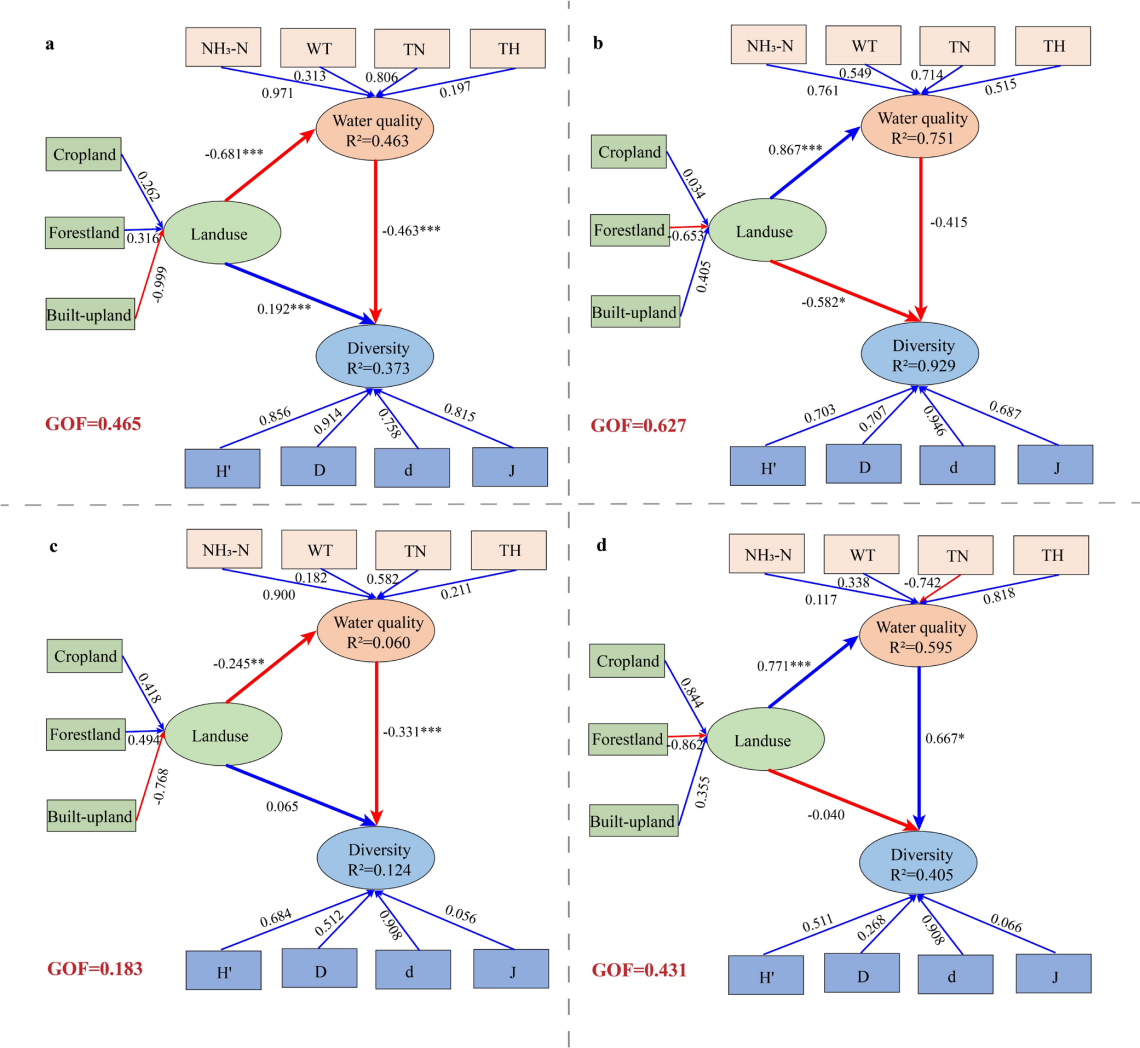
The PLS‐SEM method was used to explore the direct and indirect effects of land use structure and water quality factors on the diversity index of macrobenthic fauna. Panel (a) represents the overall model for the Lake Chaohu Basin, whereas panels (b, c, and d) represent HDA, MDA, and LDA, respectively. The arrows in the figure indicate the path relationships between variables, with red arrows representing negative effects and blue arrows representing positive effects. The values next to the arrows are the standardized path coefficients (***p < 0.001, **p < 0.01, *p < 0.05), and the *R* ^2^ inside the boxes represents the model's explanatory power for that variable. GOF indicates the overall goodness of fit of the model.

VPA showed that all environmental factors together explained 29.8% of the variation in macroinvertebrate diversity, with land use contributing 11.1%, water quality factors accounting for only 4.3%, and the two jointly explaining 14.4% (Figure 5). To further elucidate the mechanisms of their effects, we combined the CCA results with PLS‐SEM to analyze the direct and indirect pathways of land use and water quality factors on the diversity index (Figure 6). In the overall model of the Lake Chaohu Basin (Figure 6a), the weights of cropland and forestland in the land‐use latent variable were positive, whereas that of built‐up land was negative and had the largest absolute value, indicating that built‐up land was the dominant factor affecting the land‐use score. It had a significant negative effect on water quality factors (−0.681) and a significant positive direct effect on the diversity index (0.192). In the water quality latent variable, NH_3_‐N and TN had the highest weights, making them the main components of water quality factors, and they had a significant negative impact on biodiversity (−0.463). In the MDA (Figure 6b), cropland and built‐up land had positive weights, whereas forestland had a negative weight with the largest proportion. Consequently, forestland had the highest contribution rate and exerted a significant positive effect on water quality factors (0.867) but a significant negative effect on the diversity index (−0.582). Similarly, NH_3_‐N and TN contributed the most among water quality factors, and their effect on the diversity index was negative but not significant (−0.415).

In the MDA (Figure 6c), cropland and forestland had positive weights in the land‐use latent variable, whereas built‐up land had a negative weight, indicating that built‐up land was the main negative factor influencing the land‐use score. It had a significant negative effect on water quality factors (−0.245) but a non‐significant direct effect on the diversity index (0.065). Likewise, NH_3_‐N and TN had the highest weights and exerted a significant negative effect on the diversity index (−0.331). In the LDA (Figure 6d), the weights of the land‐use latent variable were similar to those in the high‐disturbance area, with forestland as the main negative factor for land use. It had a significant negative effect on water quality factors (0.771) and a non‐significant positive effect on the diversity index (−0.040). In the water quality latent variable, TH (0.818) and WT (0.338) had higher weights than TN (−0.742), indicating that the water quality score was mainly driven by TH and WT, and exerted a significant positive driving effect on biodiversity (0.667). These differences in regional response patterns likely indicate marked variation in how ecosystems regulate and adapt to external pressures across disturbance gradients.

In summary, land use has a significant driving effect on macroinvertebrate diversity, with an influence greater than that of water quality factors. Water quality factors, in general, exert an inhibitory effect on the diversity index. Specifically, in the overall model of the Lake Chaohu Basin, as well as in HDA and MDA, water quality factors have a negative effect on diversity, whereas in LDA, water quality factors have a positive driving effect.

## 4 Discussion

### 4.1 Characteristics of Macroinvertebrate Communities in Lake Chaohu Basin

Our findings revealed that macroinvertebrates had higher density but lower biomass in LDA compared to MDA. This may be because forested areas are dominated by small, fast‐reproducing benthic species that are adapted to humus‐rich surface layers but have low individual biomass (He et al. 2021). Although large benthic animals are less abundant in cropland areas, they have larger individual biomass and are better able to utilize deep soil resources and anthropogenic organic inputs, despite being frequently disturbed by cultivation (Wei et al. 2024). In contrast, streams in the HDA are more significantly affected by the combined impacts of agricultural activities, including nutrient runoff from fertilizers, pesticide pollution, and hydromorphological alterations because of land reclamation. These factors lead to more severe pollution, ultimately resulting in lower macroinvertebrate density and biomass. These findings suggest that areas dominated by cropland and forestland support richer macroinvertebrate resources and exhibit better ecological conditions than those dominated by built‐up land. Pollution‐tolerant taxa such as *Limnodrilus*, *Hippeutis*, and *Glyptotendipes* dominated the HDA, likely because of their strong stress tolerance traits—including adaptations to low oxygen, efficient utilization of eutrophic resources, and high reproductive capacity—which enable them to thrive under adverse environmental conditions such as eutrophication, organic pollution, and low dissolved oxygen levels (Karima 2021; Shi et al. 2024). In the MDA, land use is primarily agricultural, with frequent fertilizer application leading to eutrophication, creating an environment characterized by low oxygen and organic matter deposition (Shi et al. 2024). This environment promotes the dominance of moderately pollution‐tolerant gastropods (e.g., *Radix* and *Parafossarulus*) in the formation of benthic communities (Koopman et al. 2016). These species, through adaptive traits such as dual respiration (lungs and gills), omnivorous feeding habits, and moderate reproductive strategies, are able to thrive in this environment (Zhang et al. 2022). LDA are dominated by sensitive taxa, such as *Heptagenia* and *Bellamya*, which are typically found in clean, gravelly habitats with high dissolved oxygen and are highly sensitive to water quality fluctuations (Brooks and Cumming 2022; Fu et al. 2021; Kondratieff 2009).

### 4.2 Impacts of Land Use on Water Quality

Our findings indicate marked differences in how land use influences water quality among disturbance areas in the Chaohu Basin. In HDA, where built‐up land expansion predominates, large volumes of domestic and industrial wastewater are discharged into water bodies, causing eutrophication (Gani et al. 2023). In MDA, where the farmland proportion is higher, fertilizers and pesticides enter water bodies via surface runoff, degrading water quality and indirectly damaging macroinvertebrate habitats. This aligns with Pappalardo et al. (2022) and Rothwell et al. (2010), who reported that agricultural expansion exacerbates nutrient accumulation. In comparison, LDA experiences minimal human impact, retains high water quality, and favors biodiversity maintenance. Nevertheless, in several forest‐dominated low disturbance sites, we recorded relatively high nitrate concentrations, probably because of spring sampling coinciding with the forest leaf turnover period, during which abundant litterfall is decomposed by soil microbes into nitrate, leading to increased nitrogen levels in the soil.

### 4.3 Effects of Land Use and Water Quality on Macroinvertebrates

This study, through VPA, found that the independent contribution of land use was significantly higher than that of water quality factors, indicating that land use has a more prominent overall impact on biodiversity. PLS‐SEM analysis further revealed that land use not only has a direct effect on the diversity index but also indirectly affects biodiversity by influencing water quality factors. Our findings align with the results of earlier studies. For example, in the Piedmont ecoregion of North Carolina, USA, increasing urban development and impervious surface coverage have intensified water pollution, significantly reducing macroinvertebrate diversity, and led to the homogenization of species composition (Westman and Martin 2024). In the middle Rio das Mortes catchment, in southeastern Brazil nutrient concentrations in urban areas were significantly higher than those in agricultural and natural areas, accompanied by a marked decline in macroinvertebrate diversity (Gücker et al. 2024).

Interestingly, this study found that although built‐up land accounts for a small proportion of the total watershed area, it still plays a dominant role in influencing large benthic animal communities. Urban and industrial areas not only alter the natural characteristics of water bodies and degrade habitat structure, directly reducing benthic macroinvertebrate diversity, but also change nutrient concentrations in the water (e.g., NH_3_‐N and TN) through domestic sewage and industrial wastewater discharge, thereby affecting macroinvertebrate diversity (Gál et al. 2019; Ma et al. 2023). This is consistent with previous studies showing that eutrophication in urban rivers suppresses sensitive benthic taxa (Hu et al. 2022). This suggests that benthic communities in aquatic ecosystems—especially in rivers—are highly vulnerable to industrial and urban impacts, with even mild urbanization exerting significant effects on macroinvertebrate (Brown et al. 2009; Lynch et al. 2023). Notably, in LDA, water quality factors were positively correlated with the biodiversity index, primarily because moderate TH and WT may provide suitable habitat conditions for certain species. For example, studies have indicated that freshwater mollusks rely on sufficient calcium concentrations in the water to build shells, and the supply and mineralization of external Ca^2+^ directly affect their survival (Parveen et al. 2020; Pyron and Brown 2015). Additionally, elevated WT can boost the metabolic rates of freshwater invertebrates, influencing growth and reproduction timing, but when surpassing tolerance limits, they lead to adverse impacts (Piazza et al. 2020; Wan et al. 2024). This does not imply that increased pollutant levels are beneficial; rather, it reflects the promoting effect of specific water quality parameters under certain local environmental conditions.

### 4.4 Management Implications and Limitations

This study comprehensively analyzed the effects of changes in land use structure and responses of water quality factors on macroinvertebrate diversity. Implementing zoned management measures to improve water quality is crucial for watershed ecological protection and land use management (Randhir et al. 2001; Sun et al. 2020). The study shows that in HDA, land use has a significant impact on water quality. Efforts should focus on controlling the expansion of built‐up land and improving wastewater treatment efficiency to reduce pollutant inputs, as well as restoring aquatic ecosystem functions, for example, by introducing wastewater treatment upgrades with enhanced phosphorus removal and nitrification processes (Jarvie et al. 2025). In the LDA, it is necessary to balance agricultural production and ecological conservation to maintain their positive regulatory effects on the aquatic environment and to prevent biodiversity loss caused by land use degradation. In general, ecological protection and management strategies should be tailored to regional land‐use structures, water quality sensitivity, and ecosystem carrying capacity, in order to achieve both land‐use optimization and the maintenance of watershed aquatic biodiversity (Li, Zhang, Altermatt, et al. 2023; Li, Zhang, Zhang, et al. 2023; Wang et al. 2024). In addition, separate PLS‐SEM models were constructed for LDA, MDA, and HDA to compare the influence mechanisms of land use and water quality on macroinvertebrate diversity across different disturbance gradients. This approach provides targeted insights for watershed ecological management. However, this study still has certain limitations. First, the classification of disturbance intensity zones was based solely on land use type, without integrating other sources of disturbance such as landscape configuration and human activity intensity, which may affect the accuracy of the zoning and the comprehensiveness of ecological interpretation. Secondly, this study was based on a single temporal scale of observational data, which limited its ability to capture seasonal variations, long‐term trends, and potential time‐lag effects in the relationship between water quality and benthic communities.

## 5 Conclusions

Our findings indicate that land use and water quality factors together influence the community structure and biodiversity patterns of macroinvertebrates in the Lake Chaohu Basin across varying disturbance levels. Compared with water quality factors, land use had a markedly stronger explanatory power for diversity variation, with built‐up land and cropland significantly affecting community composition via both direct and indirect effects. The effects of water quality factors were spatially heterogeneous: nitrogen‐related indicators significantly suppressed diversity in HDA and MDA, whereas TH and WT promoted diversity in LDA. The findings highlight that optimizing land use structure and improving water quality are essential strategies for enhancing biodiversity and safeguarding watershed ecosystem health. Future efforts should strengthen pollution control and ecological restoration to achieve sustainable management of regional aquatic ecosystems.

## Author Contributions

**Bingling Chen:** conceptualization (equal), data curation (lead), formal analysis (lead), investigation (lead), methodology (lead), visualization (lead), writing – original draft (lead). **Qing Ji:** data curation (supporting), writing – review and editing (supporting). **Youru Yao:** data curation (supporting), writing – review and editing (supporting). **Zhiming Zhang:** investigation (lead), project administration (lead), resources (lead), supervision (lead), writing – review and editing (equal). **Yuesheng Lin:** data curation (supporting), writing – review and editing (supporting).

## Conflicts of Interest

The authors declare no conflicts of interest.

## Supporting information


**Appendix S1:** ece372415‐sup‐0001‐AppendixS1.xlsx.

**Appendix S2:** ece372415‐sup‐0002‐AppendixS2.xlsx.

**Appendix S3:** ece372415‐sup‐0003‐AppendixS3.txt.

**Appendix S4:** ece372415‐sup‐0004‐AppendixS4.docx.

**Appendix S5:** ece372415‐sup‐0005‐AppendixS5.xlsx.

## Data Availability

The data and R code supporting the findings of this study are available in the Dryad Digital Repository at https://doi.org/10.5061/dryad.g4f4qrg3m.
