# Supplementary material for: Impacts of Land Use and Water Quality on Macroinvertebrate Diversity Under Human Disturbance in the Lake Chaohu Basin, China
Source: Ecol Evol. 2025 Oct 30;15(11):e72415. doi: 10.1002/ece3.72415 (PMC12572828; doi:10.1002/ece3.72415)
Supplement: Supplementary file 4 — Appendix S4: ece372415‐sup‐0004‐AppendixS4.docx. [file ECE3-15-e72415-s004.docx]

**Impacts of Land Use and Water Quality on Macroinvertebrate Diversity under Human Disturbance in the Lake Chaohu Basin, China**

**Appendix: Supporting figures and tables**

**Classification Criteria for Disturbance Areas and Justification for K-means Clustering (k=3)**

**Definition of Low, Moderate, and High Disturbance Areas:**

Based on the percentage of each land use type within the catchment of each sampling site, the major land use categories were identified as cropland, forestland, and built-up land. The results of the three-cluster K-means analysis revealed distinct land use patterns: one group was dominated by forestland (forest cover > 70%), classified as low disturbance areas; the second group was dominated by cropland (cropland > 80%), representing moderate disturbance areas; and the third group had a significantly high proportion of impervious surfaces (>65%), defined as high disturbance areas. This classification not only exhibits a clear statistical structure but also reflects well-defined ecological differentiation, and was therefore adopted as the basis for grouping in the subsequent analyses of community structure, water quality variables, and biodiversity indices. A summary of land use proportions and their corresponding disturbance classifications is shown in Table S1.

**Justification for Selecting k=3:**

To determine the optimal number of clusters, the silhouette method was used to evaluate clustering quality across different k values. When k = 3, the average silhouette width was 0.59, which was close to the maximum observed (0.67 at k = 7), but offered better ecological interpretability and clearer separation between clusters. Moreover, selecting three groups avoids excessive fragmentation that may reduce statistical power in subsequent analyses. Therefore, k = 3 was selected as the most appropriate number of clusters. The silhouette results supporting the selection of k = 3 are presented in Table S2.

**Major Land Use Types Considered:**

Cropland, forestland, and built-up land were identified as the major land use types based on quantitative analysis. Specifically, ArcGIS 10.8 was used to calculate the area of each land use category within the catchment of each sampling site. By comparing the proportional coverage across all sites, these three land use types consistently accounted for the largest proportions and showed clear variation among sites. Therefore, they were selected as the core variables for clustering analysis and disturbance classification.

Table S1 Proportional land use composition and cluster assignment for sampling sites

| Sampling Site | Cropland/% | Forestland/% | Shrub/% | Grassland/% | Water/% | Barren/% | Built-up land/% | Cluster |
| --- | --- | --- | --- | --- | --- | --- | --- | --- |
| 1 | 84.16 | 1.28 | 0.00 | 0.00 | 7.80 | 0.00 | 6.76 | 3 |
| 2 | 15.63 | 77.54 | 0.00 | 0.00 | 1.21 | 0.00 | 5.62 | 1 |
| 3 | 23.71 | 64.59 | 0.00 | 0.00 | 0.16 | 0.00 | 11.54 | 1 |
| 4 | 3.86 | 95.89 | 0.00 | 0.00 | 0.01 | 0.00 | 0.24 | 1 |
| 5 | 84.00 | 7.85 | 0.00 | 0.00 | 2.48 | 0.00 | 5.67 | 3 |
| 6 | 94.31 | 0.02 | 0.00 | 0.00 | 1.79 | 0.00 | 3.88 | 3 |
| 7 | 93.82 | 0.00 | 0.00 | 0.00 | 1.72 | 0.00 | 4.46 | 3 |
| 8 | 91.23 | 0.03 | 0.00 | 0.00 | 1.15 | 0.00 | 7.59 | 3 |
| 9 | 94.74 | 0.00 | 0.00 | 0.00 | 1.42 | 0.00 | 3.83 | 3 |
| 10 | 80.19 | 4.82 | 0.00 | 0.00 | 1.47 | 0.00 | 13.52 | 3 |
| 11 | 77.97 | 0.91 | 0.00 | 0.01 | 13.35 | 0.00 | 7.76 | 3 |
| 12 | 75.38 | 0.00 | 0.00 | 0.00 | 3.55 | 0.00 | 21.07 | 3 |
| 13 | 86.70 | 0.00 | 0.00 | 0.00 | 4.79 | 0.00 | 8.51 | 3 |
| 14 | 87.84 | 0.00 | 0.00 | 0.00 | 7.60 | 0.00 | 4.56 | 3 |
| 15 | 84.87 | 3.14 | 0.00 | 0.00 | 4.28 | 0.00 | 7.70 | 3 |
| 16 | 92.80 | 0.00 | 0.00 | 0.00 | 2.58 | 0.00 | 4.62 | 3 |
| 17 | 91.07 | 1.69 | 0.00 | 0.00 | 2.73 | 0.00 | 4.52 | 3 |
| 18 | 88.16 | 0.11 | 0.00 | 0.06 | 2.14 | 0.00 | 9.52 | 3 |
| 19 | 68.27 | 0.00 | 0.00 | 0.01 | 1.15 | 0.00 | 30.57 | 3 |
| 20 | 84.99 | 0.04 | 0.00 | 0.00 | 3.59 | 0.00 | 11.38 | 3 |
| 21 | 57.63 | 0.12 | 0.00 | 0.00 | 34.38 | 0.00 | 7.87 | 3 |
| 22 | 65.34 | 0.04 | 0.00 | 0.00 | 29.97 | 0.00 | 4.65 | 3 |
| 23 | 86.68 | 0.00 | 0.00 | 0.00 | 6.87 | 0.00 | 6.45 | 3 |
| 24 | 83.10 | 0.04 | 0.00 | 0.00 | 6.85 | 0.00 | 10.01 | 3 |
| 25 | 77.73 | 0.36 | 0.00 | 0.00 | 8.43 | 0.00 | 13.49 | 3 |
| 26 | 81.18 | 0.02 | 0.00 | 0.00 | 8.98 | 0.00 | 9.82 | 3 |
| 27 | 79.28 | 1.57 | 0.00 | 0.02 | 15.51 | 0.00 | 3.62 | 3 |
| 28 | 25.68 | 0.00 | 0.00 | 0.00 | 13.78 | 0.00 | 60.54 | 2 |
| 29 | 86.63 | 4.96 | 0.00 | 0.44 | 1.92 | 0.00 | 6.05 | 3 |
| 30 | 91.56 | 0.00 | 0.00 | 0.00 | 1.62 | 0.00 | 6.83 | 3 |
| 31 | 11.43 | 0.00 | 0.00 | 0.00 | 2.37 | 0.00 | 86.19 | 2 |
| 32 | 7.21 | 0.00 | 0.00 | 0.00 | 0.37 | 0.00 | 92.42 | 2 |
| 33 | 50.12 | 0.00 | 0.00 | 0.00 | 30.25 | 0.00 | 19.63 | 3 |
| 34 | 67.75 | 0.00 | 0.00 | 0.00 | 27.72 | 0.00 | 4.52 | 3 |
| 35 | 91.25 | 0.00 | 0.00 | 0.00 | 1.55 | 0.00 | 7.20 | 3 |
| 36 | 89.61 | 0.00 | 0.00 | 0.00 | 1.86 | 0.00 | 8.53 | 3 |
| 37 | 92.70 | 0.00 | 0.00 | 0.00 | 4.96 | 0.00 | 2.35 | 3 |
| 38 | 11.43 | 0.00 | 0.00 | 0.00 | 2.37 | 0.00 | 86.19 | 2 |
| 39 | 93.10 | 0.00 | 0.00 | 0.00 | 2.46 | 0.00 | 4.44 | 3 |
| 40 | 89.78 | 0.40 | 0.00 | 0.00 | 4.14 | 0.00 | 5.67 | 3 |
| 41 | 69.78 | 25.18 | 0.00 | 0.00 | 1.74 | 0.00 | 3.30 | 3 |
| 42 | 85.53 | 7.32 | 0.00 | 0.00 | 1.28 | 0.00 | 5.87 | 3 |
| 43 | 74.14 | 0.00 | 0.00 | 0.00 | 1.05 | 0.00 | 24.81 | 3 |
| 44 | 58.71 | 3.94 | 0.00 | 0.00 | 32.41 | 0.00 | 4.94 | 3 |
| 45 | 12.01 | 87.78 | 0.00 | 0.01 | 0.02 | 0.00 | 0.18 | 1 |
| 46 | 0.27 | 99.73 | 0.00 | 0.00 | 0.00 | 0.00 | 0.00 | 1 |
| 47 | 0.77 | 99.22 | 0.00 | 0.00 | 0.00 | 0.00 | 0.01 | 1 |
| 48 | 3.54 | 96.27 | 0.00 | 0.00 | 0.00 | 0.00 | 0.18 | 1 |
| 49 | 4.40 | 95.14 | 0.00 | 0.00 | 0.00 | 0.00 | 0.46 | 1 |
| 50 | 25.36 | 69.33 | 0.00 | 0.00 | 0.10 | 0.00 | 5.20 | 1 |
| 51 | 14.57 | 82.64 | 0.00 | 0.00 | 0.09 | 0.00 | 2.69 | 1 |
| 52 | 32.24 | 66.38 | 0.00 | 0.00 | 0.30 | 0.00 | 1.08 | 1 |
| 53 | 96.22 | 0.06 | 0.00 | 0.00 | 1.54 | 0.00 | 2.18 | 3 |
| 54 | 96.27 | 0.59 | 0.00 | 0.00 | 1.28 | 0.00 | 1.86 | 3 |
| 55 | 49.28 | 48.39 | 0.00 | 0.00 | 0.41 | 0.00 | 1.92 | 1 |
| 56 | 90.51 | 0.04 | 0.00 | 0.00 | 4.48 | 0.00 | 4.97 | 3 |
| 57 | 82.64 | 0.17 | 0.00 | 0.00 | 0.52 | 0.00 | 16.67 | 3 |
| 58 | 80.59 | 15.89 | 0.00 | 0.00 | 0.98 | 0.00 | 2.54 | 3 |
| 59 | 90.25 | 3.07 | 0.00 | 0.00 | 2.72 | 0.00 | 3.96 | 3 |
| 60 | 93.50 | 0.05 | 0.00 | 0.00 | 2.41 | 0.00 | 4.04 | 3 |
| 61 | 93.66 | 0.19 | 0.00 | 0.00 | 2.86 | 0.00 | 3.29 | 3 |
| 62 | 91.76 | 0.35 | 0.00 | 0.00 | 1.97 | 0.00 | 5.93 | 3 |
| 63 | 93.99 | 0.77 | 0.00 | 0.00 | 1.61 | 0.00 | 3.63 | 3 |
| 64 | 62.73 | 0.00 | 0.00 | 0.02 | 7.29 | 0.00 | 29.95 | 3 |
| 65 | 18.99 | 0.00 | 0.00 | 0.01 | 0.37 | 0.00 | 80.62 | 2 |
| 66 | 94.45 | 0.00 | 0.00 | 0.00 | 0.65 | 0.00 | 4.90 | 3 |
| 67 | 29.84 | 0.01 | 0.00 | 0.00 | 24.20 | 0.02 | 45.93 | 2 |
| 68 | 85.39 | 7.09 | 0.00 | 0.00 | 2.59 | 0.00 | 4.93 | 3 |
| 69 | 69.33 | 28.93 | 0.00 | 0.00 | 0.56 | 0.00 | 1.18 | 3 |
| 70 | 58.44 | 39.69 | 0.00 | 0.00 | 0.14 | 0.00 | 1.72 | 3 |
| 71 | 95.63 | 0.00 | 0.00 | 0.00 | 1.66 | 0.00 | 2.71 | 3 |
| 72 | 92.37 | 1.29 | 0.00 | 0.08 | 0.69 | 0.00 | 5.58 | 3 |
| 73 | 86.94 | 4.51 | 0.00 | 0.00 | 4.86 | 0.00 | 3.70 | 3 |
| 74 | 84.70 | 0.00 | 0.00 | 0.00 | 1.69 | 0.00 | 13.61 | 3 |
| 75 | 79.83 | 0.00 | 0.00 | 0.00 | 2.37 | 0.00 | 17.80 | 3 |
| 76 | 92.22 | 0.00 | 0.00 | 0.00 | 2.04 | 0.00 | 5.74 | 3 |
| 77 | 76.83 | 12.86 | 0.00 | 0.05 | 1.28 | 0.00 | 8.98 | 3 |
| 78 | 92.48 | 0.26 | 0.00 | 0.00 | 3.03 | 0.00 | 4.23 | 3 |
| 79 | 43.81 | 53.80 | 0.00 | 0.00 | 0.53 | 0.00 | 1.86 | 1 |
| 80 | 84.58 | 5.42 | 0.00 | 0.19 | 1.91 | 0.00 | 7.90 | 3 |
| 81 | 87.01 | 0.00 | 0.00 | 0.00 | 8.03 | 0.00 | 4.96 | 3 |
| 82 | 87.07 | 0.00 | 0.00 | 0.00 | 5.72 | 0.00 | 7.20 | 3 |
| 83 | 85.40 | 0.00 | 0.00 | 0.00 | 10.26 | 0.00 | 4.34 | 3 |
| 84 | 91.51 | 0.05 | 0.00 | 0.00 | 6.46 | 0.00 | 1.98 | 3 |
| 85 | 90.40 | 0.00 | 0.00 | 0.00 | 7.97 | 0.00 | 1.63 | 3 |
| 86 | 63.04 | 4.13 | 0.00 | 0.02 | 21.89 | 0.00 | 10.93 | 3 |
| 87 | 90.13 | 0.00 | 0.00 | 0.00 | 3.91 | 0.00 | 5.97 | 3 |
| 88 | 75.12 | 0.00 | 0.00 | 0.00 | 0.03 | 0.00 | 24.84 | 3 |
| 89 | 37.97 | 0.00 | 0.00 | 0.05 | 2.65 | 0.01 | 59.32 | 2 |
| 90 | 89.68 | 0.00 | 0.00 | 0.00 | 3.84 | 0.00 | 6.48 | 3 |
| 91 | 60.44 | 32.65 | 0.00 | 0.01 | 5.80 | 0.00 | 1.10 | 3 |
| 92 | 86.44 | 0.00 | 0.00 | 0.00 | 3.33 | 0.00 | 10.23 | 3 |
| 93 | 4.61 | 94.62 | 0.01 | 0.06 | 0.01 | 0.00 | 0.69 | 1 |
| 94 | 69.70 | 0.02 | 0.00 | 0.01 | 1.88 | 0.00 | 28.39 | 3 |
| 95 | 6.34 | 93.57 | 0.00 | 0.00 | 0.00 | 0.00 | 0.09 | 1 |
| 96 | 56.77 | 38.41 | 0.00 | 0.00 | 1.37 | 0.00 | 3.46 | 3 |
| 97 | 38.68 | 52.93 | 0.00 | 0.00 | 2.25 | 0.00 | 6.15 | 1 |
| 98 | 31.86 | 65.72 | 0.00 | 0.02 | 0.80 | 0.00 | 1.61 | 1 |
| 99 | 90.54 | 0.00 | 0.00 | 0.00 | 4.58 | 0.00 | 4.88 | 3 |
| 100 | 65.34 | 0.04 | 0.00 | 0.00 | 29.97 | 0.00 | 4.65 | 3 |
| 101 | 84.90 | 11.65 | 0.00 | 0.00 | 1.09 | 0.00 | 2.35 | 3 |
| 102 | 90.19 | 2.28 | 0.00 | 0.00 | 0.78 | 0.00 | 6.75 | 3 |
| 103 | 68.66 | 22.01 | 0.00 | 0.00 | 3.66 | 0.00 | 5.66 | 3 |
| 104 | 20.35 | 77.47 | 0.00 | 0.00 | 0.29 | 0.00 | 1.88 | 1 |
| 105 | 89.03 | 4.80 | 0.00 | 0.00 | 0.98 | 0.00 | 5.19 | 3 |
| 106 | 92.05 | 0.00 | 0.00 | 0.00 | 3.83 | 0.00 | 4.12 | 3 |
| 107 | 69.78 | 25.18 | 0.00 | 0.00 | 1.74 | 0.00 | 3.30 | 3 |
| 108 | 47.71 | 46.41 | 0.00 | 0.00 | 0.85 | 0.00 | 5.04 | 1 |
| 109 | 94.11 | 0.02 | 0.00 | 0.00 | 3.29 | 0.00 | 2.58 | 3 |
| 110 | 91.31 | 0.25 | 0.00 | 0.00 | 6.10 | 0.00 | 2.34 | 3 |
| 111 | 86.60 | 6.15 | 0.00 | 0.05 | 3.18 | 0.00 | 4.02 | 3 |
| 112 | 68.46 | 20.55 | 0.00 | 0.00 | 0.67 | 0.00 | 10.31 | 3 |
| 113 | 75.86 | 14.85 | 0.00 | 0.00 | 0.31 | 0.00 | 8.98 | 3 |
| 114 | 23.75 | 73.98 | 0.00 | 0.00 | 0.02 | 0.00 | 2.26 | 1 |
| 115 | 89.87 | 0.01 | 0.00 | 0.00 | 0.42 | 0.00 | 9.70 | 3 |
| 116 | 86.75 | 0.73 | 0.00 | 0.00 | 5.90 | 0.00 | 6.63 | 3 |
| 117 | 87.00 | 6.22 | 0.00 | 0.32 | 3.11 | 0.00 | 3.35 | 3 |
| 118 | 93.44 | 0.11 | 0.00 | 0.00 | 2.83 | 0.00 | 3.61 | 3 |
| 119 | 65.74 | 3.32 | 0.00 | 0.00 | 27.67 | 0.00 | 3.27 | 3 |
| 120 | 92.72 | 1.42 | 0.00 | 0.00 | 0.53 | 0.00 | 5.32 | 3 |
| 121 | 72.99 | 11.29 | 0.00 | 0.00 | 10.45 | 0.00 | 5.26 | 3 |
| 122 | 93.27 | 0.00 | 0.00 | 0.00 | 1.19 | 0.00 | 5.55 | 3 |
| 123 | 5.57 | 91.75 | 0.00 | 0.00 | 0.00 | 0.00 | 2.68 | 1 |
| 124 | 89.01 | 1.52 | 0.00 | 0.00 | 6.57 | 0.00 | 2.90 | 3 |
| 125 | 69.69 | 6.83 | 0.00 | 0.00 | 17.60 | 0.00 | 5.88 | 3 |
| 126 | 94.55 | 0.00 | 0.00 | 0.00 | 1.34 | 0.00 | 4.12 | 3 |
| 127 | 81.85 | 0.06 | 0.00 | 0.00 | 1.18 | 0.00 | 16.91 | 3 |
| 128 | 46.55 | 0.00 | 0.00 | 0.01 | 1.31 | 0.00 | 52.13 | 2 |
| 129 | 42.29 | 57.51 | 0.00 | 0.00 | 0.08 | 0.00 | 0.11 | 1 |
| 130 | 93.16 | 0.00 | 0.00 | 0.00 | 1.97 | 0.00 | 4.87 | 3 |
| 131 | 88.32 | 0.06 | 0.00 | 0.00 | 1.95 | 0.00 | 9.67 | 3 |
| 132 | 87.75 | 5.96 | 0.00 | 0.05 | 2.48 | 0.00 | 3.77 | 3 |
| 133 | 90.02 | 0.13 | 0.00 | 0.00 | 6.95 | 0.00 | 2.90 | 3 |
| 134 | 30.02 | 68.01 | 0.00 | 0.00 | 0.35 | 0.00 | 1.61 | 1 |
| 135 | 77.96 | 11.48 | 0.00 | 0.00 | 0.00 | 0.00 | 10.56 | 3 |
| 136 | 92.26 | 2.12 | 0.00 | 0.00 | 1.61 | 0.00 | 4.00 | 3 |
| 137 | 89.01 | 0.00 | 0.00 | 0.00 | 1.62 | 0.00 | 9.38 | 3 |
| 138 | 34.38 | 0.00 | 0.00 | 0.01 | 1.62 | 0.00 | 63.99 | 2 |
| 139 | 78.07 | 0.00 | 0.00 | 0.03 | 4.64 | 0.00 | 17.26 | 3 |
| 140 | 6.82 | 90.22 | 0.00 | 0.00 | 0.00 | 0.00 | 2.97 | 1 |
| 141 | 3.69 | 95.54 | 0.00 | 0.00 | 0.00 | 0.00 | 0.78 | 1 |
| 142 | 50.01 | 0.00 | 0.00 | 0.00 | 0.24 | 0.00 | 49.75 | 2 |
| 143 | 51.87 | 27.68 | 0.00 | 0.00 | 8.74 | 0.00 | 11.71 | 3 |
| 144 | 40.39 | 58.23 | 0.00 | 0.00 | 0.14 | 0.00 | 1.24 | 1 |
| 145 | 80.84 | 2.24 | 0.00 | 0.00 | 2.93 | 0.00 | 13.99 | 3 |
| 146 | 89.98 | 3.48 | 0.00 | 0.00 | 1.61 | 0.00 | 4.92 | 3 |
| 147 | 92.50 | 0.01 | 0.00 | 0.00 | 3.54 | 0.00 | 3.95 | 3 |
| 148 | 71.47 | 0.18 | 0.00 | 0.06 | 14.45 | 0.00 | 13.84 | 3 |
| 149 | 69.33 | 28.93 | 0.00 | 0.00 | 0.56 | 0.00 | 1.18 | 3 |
| 150 | 81.75 | 0.00 | 0.00 | 0.00 | 3.81 | 0.00 | 14.44 | 3 |

Table S2 Cluster-based summary of land use proportions and disturbance classification

| Cluster | Cropland/% | Forestland/% | Shrub/% | Grassland/% | Water/% | Barren/% | Built-up land/% | Disturbance Type |
| --- | --- | --- | --- | --- | --- | --- | --- | --- |
| 1 | 20.44 | 77.03 | 0.00 | 0.00 | 0.29 | 0.00 | 2.23 | LDA |
| 2 | 27.35 | 0.00 | 0.00 | 0.01 | 4.93 | 0.00 | 67.71 | HDA |
| 3 | 82.88 | 4.23 | 0.00 | 0.01 | 5.39 | 0.00 | 7.49 | MDA |

Note: LDA: low disturbance area, MDA: moderate disturbance area; HDA: high disturbance area
